# A Vision for Cytokine Biology with 20/20 Clarity

**DOI:** 10.1093/function/zqaa042

**Published:** 2020-12-26

**Authors:** Simon A Jones, Clare Bryant, Clare M Lloyd, Iain McInnes, Luke O’Neill

**Affiliations:** 1 Division of Infection & Immunity, The School of Medicine, Cardiff University, Wales, UK; 2 Systems Immunity Research Institute, Cardiff University, Wales, UK; 3 Department of Veterinary Medicine, University of Cambridge, Cambridge, UK; 4 Faculty of Medicine, National Heart & Lung Institute, Imperial College London, London, UK; 5 Institute of Infection & Immunity, School of Medicine, Dentistry & Nursing, University of Glasgow, Glasgow, Scotland, UK; 6 School of Biochemistry & Immunology, Trinity College Dublin, Dublin, Ireland

## Commentary

Cytokine receptor systems have evolved to sense and interpret environmental cues that affect cellular proliferation, differentiation, and survival.^1^ Here, studies describing the biology of interferons, interleukins, chemokines, growth factors, and members of the tumor necrosis factor family, showcase involvements for these proteins in both general physiology and pathophysiology.^1^ These discoveries have pioneered advances in the scientific understanding of immune function and fuelled clinical innovations that affect the diagnosis and treatment of infection, inflammation and cancer. Indeed, biological drugs and small molecule inhibitors that target specific cytokine pathways are now commonly prescribed in the treatment of cancer, infectious disease, or complex immune-mediated inflammatory diseases. These include drugs against tumor necrosis factor (TNF)α, interleukin (IL)-6, IL-17, granulocyte-macrophage colony-stimulating factor (GM-CSF), recombinant cytokine modalities such as type-1 interferon, and oral inhibitors that block cytokine signaling intermediates (eg, Janus kinases).^2^

The introduction of cytokine targeting therapies in routine clinical practice has revolutionized the treatment of patients with chronic diseases. However, there is still much to be learnt about the biological properties of these proteins. While early advances in the development of these therapies arose from discoveries made in animal models of disease, clinical experience with cytokine-targeting drugs has identified new and exciting roles for cytokines in metabolism, mental health, fatigue, sleep, and immune homeostasis.^2^ These developments have pioneered research in immunometabolism, psychoneuroimmunology, and multimorbidity.^3^^,^^4^ The efficacy of biological drugs and small molecule inhibitors in clinical practice has also been instrumental in advancing personalized approaches for patient care. Patients often show inadequate responses to certain classes of therapy and patients with the same underlining condition frequently display varying efficacies to inhibitors of TNFα, IL-6, or IL-17.^2^ It is, therefore, necessary to understand the molecular and cellular nodes that promote the maintenance of cytokine-driven pathology in patients with complex diseases. This patient-centric approach echoes the philosophy of Sir William Osler (1849–1919) who advocated that “The good physician treats the disease; the great physician treats the patient who has the disease.” Such teachings are pertinent to the research conducted in the field of cytokine biology where new technologies are now allowing a more informed appreciation of how cytokine signals contribute to the narrative of the disease. For example, single-cell methodologies, combined with genetic and functional genomics, offer exciting opportunities to identify how the cytokine network instructs the course of the disease. These types of holistic analysis providing information on the keystone cytokines responsible for maintaining the architecture of the inflammatory response in disease, and that may be tractable for clinical benefit. Advances in precision medicine further illustrate the importance of monitoring cytokines and cytokine receptor signals as diagnostic or prognostic indicators of disease severity. Thus, cytokine responses act as barometers of health and disease and are increasingly investigated to predict responses to therapy or the most appropriate therapy for a patient or patient group. So, what is the future for cytokine research?

Talk to any cytokine biologist and each will have their favorite. As scientists, we often show emotional attachments to an individual cytokine or cytokine family. However, standing back for this bias, there are clear areas of common interest emerging within the field. Some selected examples for future endeavor include:

## Fine-Tuning Cytokine Receptor Signals for Therapy

Structure-function studies and investigations of cytokine receptor signaling have opened possibilities to alter the bioactivity or potency of cytokines for therapy.^5^ Here, engineering technologies allow cytokine activities to be modified leading to the generation of super-agonists, partial agonists, and antagonists. These derivatives offer therapeutic opportunities to fine-tune cytokine activities in health and disease. Notable examples include members of the IL-1 (eg, EBI-005, Rilonacept), IL-2 (eg, Super-IL-2; H9, H9-RETR, H9-RET, BiG, ALT-803), IL-6 (eg, olamkicept, Hyper-IL-6, IC7Fc), IL-10 (eg, Pegilodekakin, R5A11D), and IL-12 (eg, P40 homodimer) cytokine superfamilies. Here, encouraging data using some of these agents in oncology trials demonstrate the power of these strategies and showcase their potential in the treatment of cancer, immune-mediated inflammatory diseases, allergy, and immunodeficiencies.^5^ These strategies offering new opportunities to develop standard alone drug interventions or therapies that work in combination with more established drug interventions.

## The Identification of Biological Rheostats of Cytokine Signaling–

The idea that cytokine receptor signaling remains fixed has changed over the last 15 years. It is now accepted that the signaling cascades employed by cytokines are highly dynamic and subject to multiple forms of regulation. These mechanisms extend beyond traditional negative-feedback inhibitors (eg, SOCS proteins, IKB, Smad6) of cytokine signaling. Notable examples include regulatory mechanisms that act on phosphorylation events immediately following cytokine receptor engagement. Such systems moderate the balance of transcription factor activation and shape the transcriptional output of a cytokine. This form of regulation is particularly evident in receptor systems employing the Jak-STAT pathway.^6^ Here, protein tyrosine phosphatases involved in metabolic and immune regulation often control kinases and transcription factors in the signaling cascade.^6–8^

Cytokines receptors that signal through the Jak-STAT pathway typically engage a select panel of STAT transcription factors. The transcriptional properties of these proteins are highly complex, and they often work in cooperation or through cross-regulatory mechanisms.^6^ Here, genetic ablation studies show that STAT1-activating cytokines display elevated STAT3 responses in the absence of STAT1. A similar relationship is also seen between STAT5 and STAT6. Physiological processes (eg, protein tyrosine phosphatases) that act on the immediate activation of STAT transcription factors are likely to elicit similar outcomes.^8^ Thus, allowing cytokine responses to be tailored to the activation status of the cell type or local inflammatory environment. Such mechanisms may explain the basis of disease heterogeneity in patients with common underlining pathology and provide fresh insights into disease susceptibilities associated with genetic polymorphisms in regulatory enzymes (eg, *PTPN2*, *PTPN22*).

## Cytokines in Comorbidity and Psychoneuroimmunology

Animal models supporting the translational development of anti-cytokine and anti-cytokine receptor blockers significantly advanced our understanding of how biological drugs and small molecule inhibitors target cytokines for clinical benefit. However, clinical experience shows that these therapies elicit broader benefits beyond the control of inflammation and tissue pathology. These include improvements in cardiovascular risk, anemia, metabolism (eg, glucose, lipid, iron), fatigue, sleep, and mental well-being. Combining experimental medicine approaches and mechanistic investigations in model systems, researchers have begun to tease apart the biology behind these comorbidities. For example, the cytokine regulation of hepcidin linked with iron metabolism, anemia, and fatigue, and impacts on endothelial dysfunction, adhesion, clot formation, and vascular tone relevant to cardiovascular risk. Others are currently more observational and include correlations with patterns of sleep, alternations in neuroendocrine function, and circadian regulation. The links between immunology and neuroscience are particularly striking. Here, the ability of biological drugs to alter depression, anxiety, anhedonia, and mood is increasingly relevant for patients combating chronic disease. Estimates suggest that 30%–40% of patients display symptoms that impact their psychological well-being and overall quality of life. To support the diagnosis and treatment of psychopathology in chronic disease; it is necessary to understand why some patients are more susceptible to these complications. Here, acute psychosocial stress and depression, and feelings of fatigue, insomnia, and anger often correlate with changes in inflammatory cytokines (eg, TNF, IL-1, IL-6).^9^ These studies have led to new hypotheses specific to the development of psychopathology. For example, alterations in endothelial blood-brain barrier function or metabolic dysfunction. A major challenge is to define how cytokine networks instruct the regulation of these pathways in depression or psychopathology. Measures observed in response to acute stimuli (eg, Type 1 interferons, lipopolysaccharide) have provided some fresh insights, but these ideas require further evaluation in the context of chronic inflammatory disease. This will necessitate new collaborations between neuroscientists, immunologists, clinicians, and geneticists.

## Cytokines in Pain

Patients with long-term chronic illnesses often struggle with debilitating pain as a consequence of their disease. A failure to appropriately manage pain significantly affects patient quality of life and their ability to maintain a sustainable work–life balance. In conditions where tissue damage drives localized pain, inflammatory processes promote nociception in small nerve fibers. These inflammatory processes may reflect classical cytokine-mediated stress responses to injury or trauma leading to activation of Jun N-terminal kinase (JNK), extracellular signal-regulated kinases (ERK), and p38 mitogen-activated protein kinase (MAPK).^10^ Other cytokines may elicit more specific consequences in nerve cells. Certain cytokines often display neurotrophic properties essential for normal physiology. Under chronic disease, these cytokine networks become disrupted, and the breakdown in normal tissue homeostasis gives rise to a heightened form of pain perception. Thus, a neuroinflammation of the somatosensory nervous system would drive chronic neuropathic pain.^10^ Here, clinical investigations of cytokine blocking therapies in patients and corresponding mouse studies provide evidence of the link between pain and cytokines. Data from clinical trials show that biological drugs targeting TNF, IL-1, IL-6, and IL-17 suppress indices of pain. Mouse studies of pain in chronic disease also demonstrate links to IL-23 and GM-CSF. We now need to understand the mechanisms governing cytokine-mediated pain. Clinical evidence from trials of baricitinib and tofacitinib in rheumatoid arthritis patients show that oral inhibitors of the Jak-STAT pathway substantially improve patient outcome measures of pain. However, pain is likely controlled at multiple levels suggesting the need for biomarkers of cytokine activity that can be related to patient outcome measures of pain.^10^ Thus, advances in cytokine biology are likely to emerge together with new clinical innovations supporting the management of pain in patients with chronic disease.

## Cytokines in Immune and Tissue Homeostasis

Cytokines often display hormone-like characteristics. These include physiological activities governing vascular tone, lipid metabolism, insulin resistance, mitochondrial bioenergetics, and neuroendocrine function. As a consequence, cytokines instruct processes that affect tissue and immune homeostasis.

Various mechanisms have evolved to safeguard the appropriate regulation of cytokine production during normal physiology. Here, cytokine gene expression is controlled by microRNAs (eg, let-7a), RNA-binding proteins (eg, Lin28B, Arid5a), RNases (eg, regnase-1), circadian control factors (eg, Per1), and genomic super-enhancers. While our understanding of these systems has increased in the past decade, it is important to establish how these processes become altered to affect physiology or pathophysiology. For example, cytokines control epithelial turnover, bone mass and architecture, liver regeneration, and sleep. In pregnancy, cytokines also regulate gametogenesis, uterine receptivity, placental implantation, embryogenesis, and fetal development. Identifying how cytokines shape these processes will generate new insights into the pathogenesis of conditions such as pre-eclampsia, recurrent miscarriage, endometriosis, hypertension, fibrosis, or rare connective tissue disorders.

These studies will also improve our assessment of contraindications that occur following treatment with biological drugs or small molecule inhibitors that block cytokine pathways essential for normal physiology. Notable examples include neutropenia, anemia, and increased risk of infections at epithelial or mucosal surfaces (eg, the urinary and gastrointestinal tracts).^2^ These are illustrated by contraindications associated with tocilizumab and other IL-6 targeting therapies. These drugs are often less effective in diseases where IL-6 contributes to the maintenance of epithelial homeostasis or barrier integrity. Consequently, patients with a history of gastric perforations or associated diverticulitis should avoid therapies that block IL-6 or its receptor cassette. These studies emphasize the need to predict the involvement of a drug-targeted cytokine in the pathophysiology using criteria that maximize patient response rates.

In conclusion, the field of cytokine biology remains vibrant and intellectually challenging. Research in this area continues to align fundamental discovery science with clinical translation and innovation. Here, cytokine biology will have a significant influence on the advancement of precision medicine and will be increasingly applied to support the diagnosis, stratification, and treatment of patients with complex chronic diseases. Rose-tinted glasses? Let’s see.

## Conflict of Interest Statement

The authors are lead organizers of Cytokines2021; the ninth *International Cytokine & Interferon Society* conference (City Hall, Cardiff, October 17–20, 2021). S.A.J. has received research support (GSK, Mestag Therapeutics, Hoffmann la Roche, NovImmune SA, Ferring Pharmaceuticals) and consultancy (Sanofi-Regeneron, GSK, Janssen, EUSA Pharmaceuticals, NovImmune SA., Chugai Pharmaceutical, Hoffmann la Roche) from pharmaceutical companies and small-medium enterprises involved in biological drug development. C.B. has received grant funding from Apollo therapeutics and is a scientific consultant for Nadthera and Lightcast. C.M.L. declares no conflicts of interest. I.B.M. has received honoraria or research funding from the following companies who make cytokine targeting agents (Abbvie, AstraZeneca, GSK, Lilly, Pfizer, BMS, UCB, Gilead, Janssen, Novartis). L.O. declared no conflicts of interest.
